# Supplementary material for: 4/4 and more, rhythmic complexity more strongly predicts groove in common meters
Source: Commun Psychol. 2025 Dec 16;3:185. doi: 10.1038/s44271-025-00360-0 (PMC12708351; doi:10.1038/s44271-025-00360-0)
Supplement: Supplementary file 2 — Supplementary Material [file 44271_2025_360_MOESM2_ESM.pdf]

Supplementary Materials

Title	Artist	Excerpt	Pulse Entropy	Tempo	Genre	Time Signature	Meter
South of Heaven	Slayer	1:25-1:33	0.50008	120	Metal	4/4	Common
Peggy	Orchards	0:00-0:08	0.76145	130	Alt pop	4/4	Common
Voyager	Daft Punk	0:32-0:48	0.59618	120	EDM	4/4	Common
Get It Right	Aretha Franklin	0:11-0:19	0.65258	121	Soul/funk	4/4	Common
I Wanna Be Your Lover	Prince	0:00-0:08	0.56799	117	Pop/funk	4/4	Common
I Got The Feelin	James Brown	0:00-0:03 (looped)	0.71119	114	Soul/funk	4/4	Common
What About Me	Snarky Puppy	0:15-0:23	0.7308	127	Jazz/funk	4/4	Common
Getaway	Earth, Wind & Fire	0:00-0:09	0.75177	112	Funk	4/4	Common
Smash	Avishai Cohen	0:08-0:16	0.7938	115	Jazz/ experimental	4/4	Common
Pinzin Kinzin	Avishai Cohen Trio	2:01-2:05 (looped)	0.76748	116	Jazz/ experimental	4/4	Common
The Detail	Delta Sleep	0:17-0:26	0.73743	112	Math rock	4/4	Common
Jesus Bill!	Delta Sleep	0:45-0:54	0.74524	112	Math rock	12/8 and	Uncommon

						6/8 + 5/8 + 6/8 + 9/8	
Melody 4 Riff	Tera Melos	0:52-1:00	0.7514	110	Math rock	4/4 and 7/8	Uncommon
Melody 4 Intro	Tera Melos	0:00-0:08	0.73634	110	Math rock	4/4	Common
Weeds	Hoover	0:17-0:27	0.70739	129	Post-hardcore	5/4 + 4/4 (9/4)	Uncommon
Closer	Iron Curtis	0:09-0:19	0.6743	109	Electronic	4/4	Common
No Signal	CHON	0:35-0:53	0.73545	115	Math rock	9/16 and 5/16	Uncommon
Money	Pink Floyd	0:27-0:41	0.61463	126	Classic Rock	7/4	Uncommon
Want to Come Back to My Room and Listen to Some Belle and Sebastian	This Town Needs Guns	0:05-0:13	0.71463	128	Math rock	9/4	Uncommon
Tempting Time	Animals As Leaders	0:21-0:30	0.77851	120	Progressive metal	4/4	Common
Some Kind of Game*	Against All Logic	0:00-0:08	0.62619	128	EDM	4/4	Common
1992*	no_4mat	0:52-1:00	0.68436	128	EDM	4/4	Common
Mouths Like Sidewinder	The Fall of Troy	0:06-0:13	0.79688	125	Mathcore	4/4	Common

Missiles							
Saigo No Bansan	Mouse on the Keys	0:53-1:01	0.79159	125	Jazz/ experimental	7/8	Uncommon
Música das Nuvens e do Chão	Hermeto Pascoal	1:43-1:52	0.79722	130	Jazz/samba	7/8	Uncommon
Mixing Pot	Hermeto Pascoal	1:20-1:30	0.70208	108	Jazz/samba	7/8	Uncommon
Mission Impossible*	Lalo Schifrin Orchestra	0:01-0:11	0.72977	110	Jazz	5/4	Uncommon
Take Five	Dave Brubeck (Live)	6:39-6:50	0.74477	110	Jazz	5/4	Uncommon
Blue Rondo a la Turk	Dave Brubeck	0:23-0:32	0.79042	114	Jazz	9/8	Uncommon
Billie Jean*	Michael Jackson	0:21-0:29	0.66802	117	Pop	4/4	Common
Beat It*	Michael Jackson	0:25-0:36	0.63123	130	Pop	4/4	Common
Icarus Lives	Periphery	0:09-0:17	0.75509	110	Progressive metal	4/4	Common
Get Stingy*	Partiboi69	2:53-3:01	0.62975	110	Hip-Hop/ Funk	4/4	Common
Legit Tattoo Gun	The Front Bottoms	0:00-0:8	0.62448	130	Indie rock	4/4	Common

Seven Nation Army*	White Stripes	0:59-1:07	0.64825	124	Alt Rock	4/4	Common
Unretrofied	The Dillinger Escape Plan	0:24-0:32	0.63802	120	Mathcore	4/4	Common
Midnight Carroll Street	Into It. Over It.	1:10-1:16	0.58759	130	Indie rock	4/4	Common
Walk*	Pantera	0:09-0:16	0.68916	118	Metal	4/4	Common
Nunc Stans	Cynic	0:11-0:22	0.64854	110	Metal	6/8 and 7/8	Uncommon
The Hand That Feeds	Nine Inch Nails	1:37-1:45	0.65396	128	Industrial/ Electronic	4/4	Common
Touch	I Love Your Lifestyle	0:00-0:08	0.54599	110	Emo	4/4	Common
Everyplace is a House	Maps & Atlases	0:00-0:9	0.78262	120	Math rock	4/4	Common
Into the Mirror	Minus the Bear	0:26-0:34	0.61145	118	Indie rock	4/4	Common
Lazy Eye	Silversun Pickups	0:09-0:16.5	0.6127	127	Indie rock	4/4	Common
Electric Feel	MGMT	0:09-0:16	0.66096	110	Indie rock	6/4	Uncommon
The Day I Tried To Live	Soundgarden	0:34-0:48	0.6873	130	Grunge	7/8 and 4/4	Uncommon
Schism	Tool	0:27-0:41	0.62502	110	Progressive metal	5/8 and 7/8	Uncommon
Unsquare Dance	Dave	0:13-0:24	0.75006	116	Jazz	7/8	Uncommon

	Brubeck						
Souls of Black	Testament	0:08-0:15	0.6741	129	Metal	12/8	Uncommon
Boxelder	Motion City Soundtrack	1:20-1:24 (looped)	0.58296	120	Rock	7/4	Uncommon
Panspermia	Eat Static	1:55-2:07	0.66214	114	Electronic	9/8 x 2 (18/8)	Uncommon
Trojans Low	The Algorithm	1:53-2:03	0.63065	130	EDM	4/4	Common
Trojans High	The Algorithm	0:44-0:58	0.81538	130	Electronic	4/4	Common
EDM in 78	Gabe Miller	4:55-5:02	0.63785	120	EDM	7/8	Uncommon
Five	Lamb	1:33-1:42	0.75083	110	Electronic	10/8 + 10/8 + 11/8 (+ 11/8)	Uncommon
This Is What It Feels Like	Armin van Buuren	1:39-1:51	0.59829	130	EDM	6/4 and 4/4	Uncommon
GOAT	Polyphia	1:47-1:56	0.74039	110	Progressive metal	4/4	Common
Weird Circles	Tera Melos	2:42-2:52	0.75006	114	Math rock	5/4	Uncommon
Mr. Beat	King Gizzard and the Lizard Wizard	0:15-0:27	0.67041	127	Rock	7/4	Uncommon
Five Sticks	Led Zeppelin	0:00-0:09	0.78264	110	Classic Rock	5/8 (x4) +and 6/8	Uncommon

						(x2)	
Estimated Prophet	The Grateful Dead	0:01-0:10	0.73284	128	Classic Rock	7/8	Uncommon
2+2=5*	Radiohead	0:16-0:26	0.65048	130	Alternative Rock	7/4	Uncommon
Don't Eat The Yellow Snow Suite	Frank Zappa	0:15-0:25	0.70487	130	Jazz Rock	7/4	Uncommon

Table 1. Detailed information about each musical clip in the study. Songs where tempo was manipulated are denoted with asterisks.

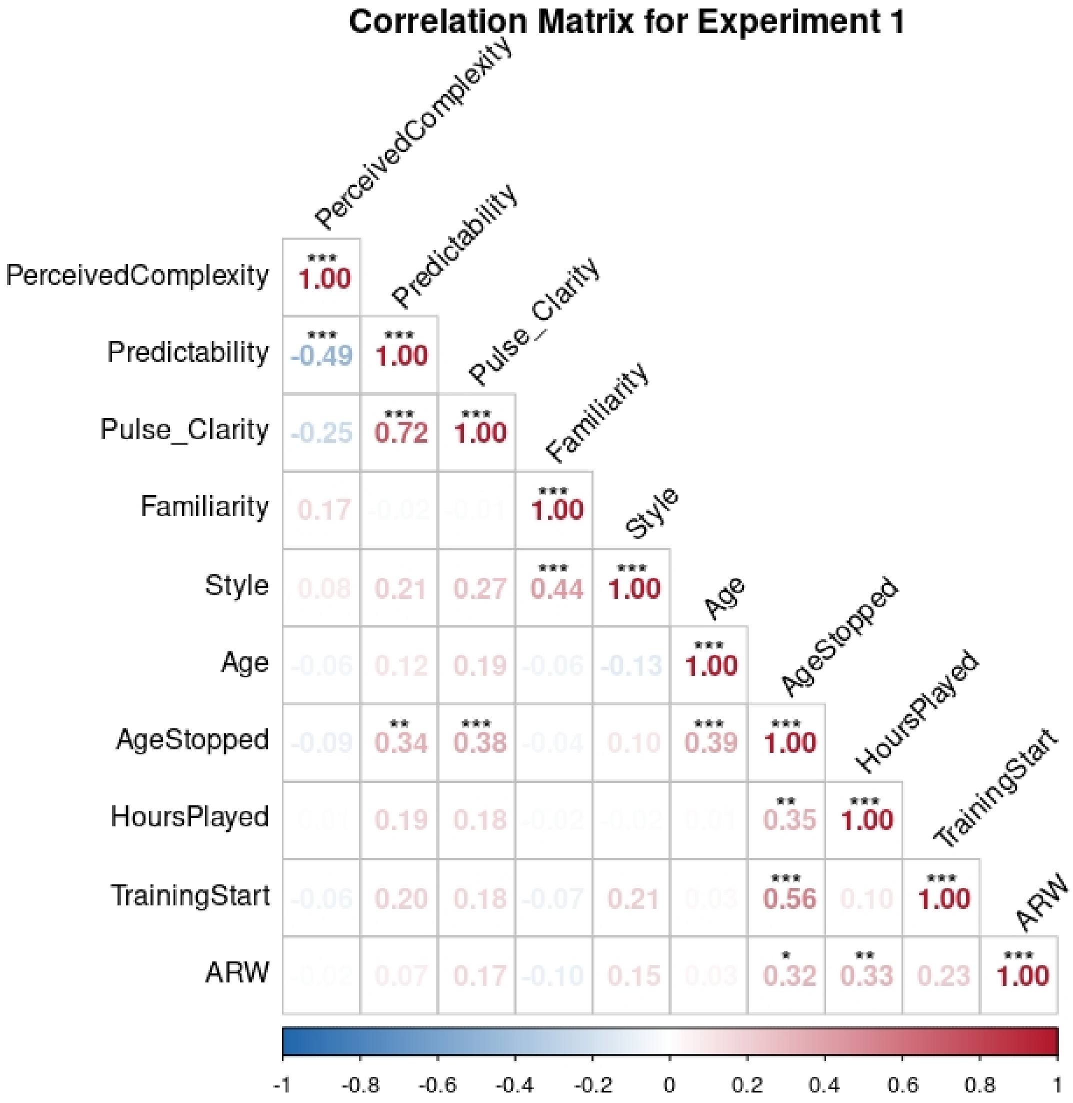

Supplementary Figure 1. Correlation matrix of all measured variables in Experiment 1. “AgeStopped” refers to the age that participants stopped playing music (set to current age if they are still playing music), “HoursPlayed” refers to the reported hours of music playing per week, “TrainingStart” refers to the age that formal musical training started (set to zero if they never started formal musical training), and “ARW” refers to the overall musical reward sensitivity obtained from the Barcelona Musical Reward Questionnaire. Bonferroni-corrected alpha values at $p < 0.05$, $p < 0.01$, and $p < 0.001$ are denoted with single, double, and triple asterisks.

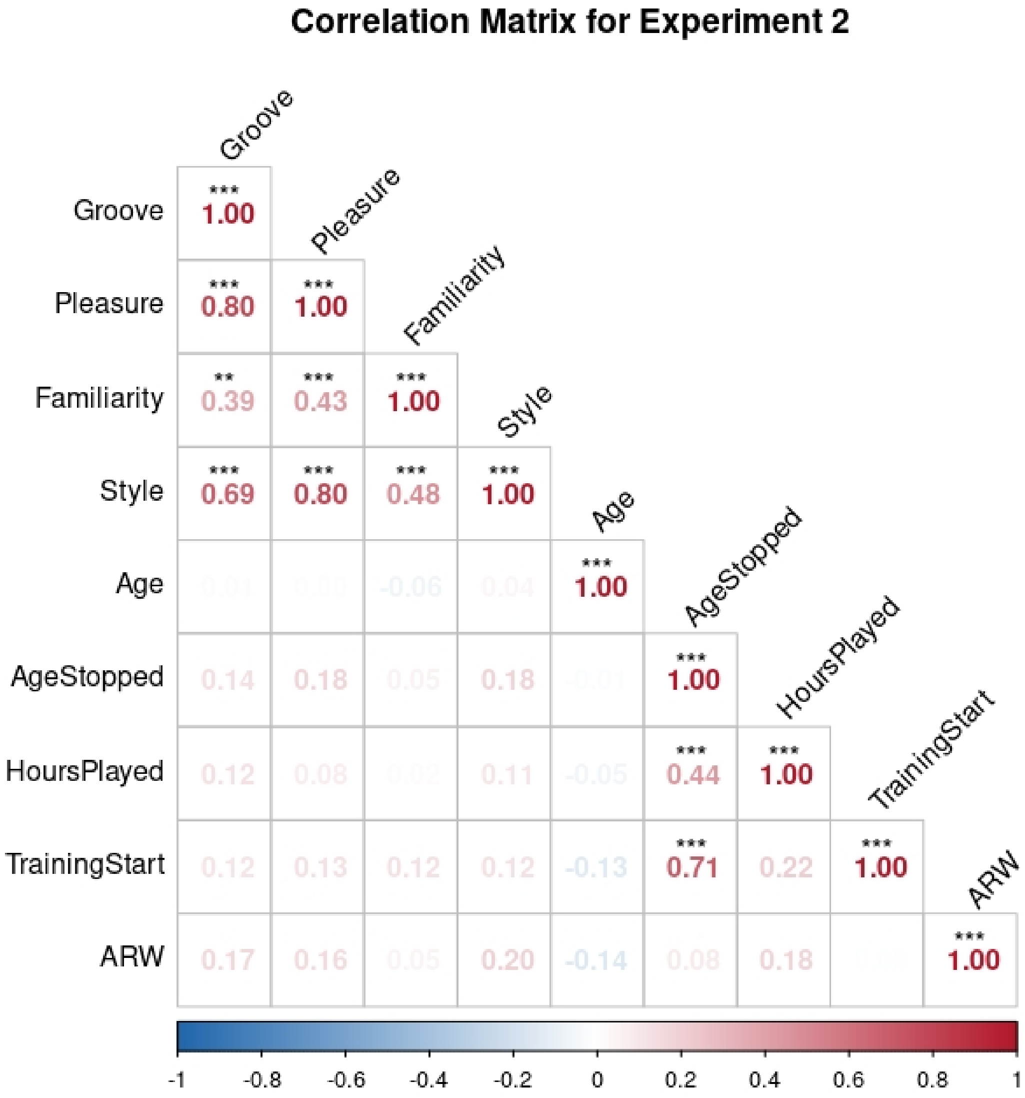

Supplementary Figure 2. Correlation matrix of all measured variables in Experiment 2. “AgeStopped” refers to the age that participants stopped playing music (set to current age if they are still playing music), “HoursPlayed” refers to the reported hours of music playing per week, “TrainingStart” refers to the age that formal musical training started (set to zero if they never started formal musical training), and “ARW” refers to the overall musical reward sensitivity obtained from the Barcelona Musical Reward Questionnaire. Bonferroni-corrected alpha values at $p < 0.05$, $p < 0.01$, and $p < 0.001$ are denoted with single, double, and triple asterisks.

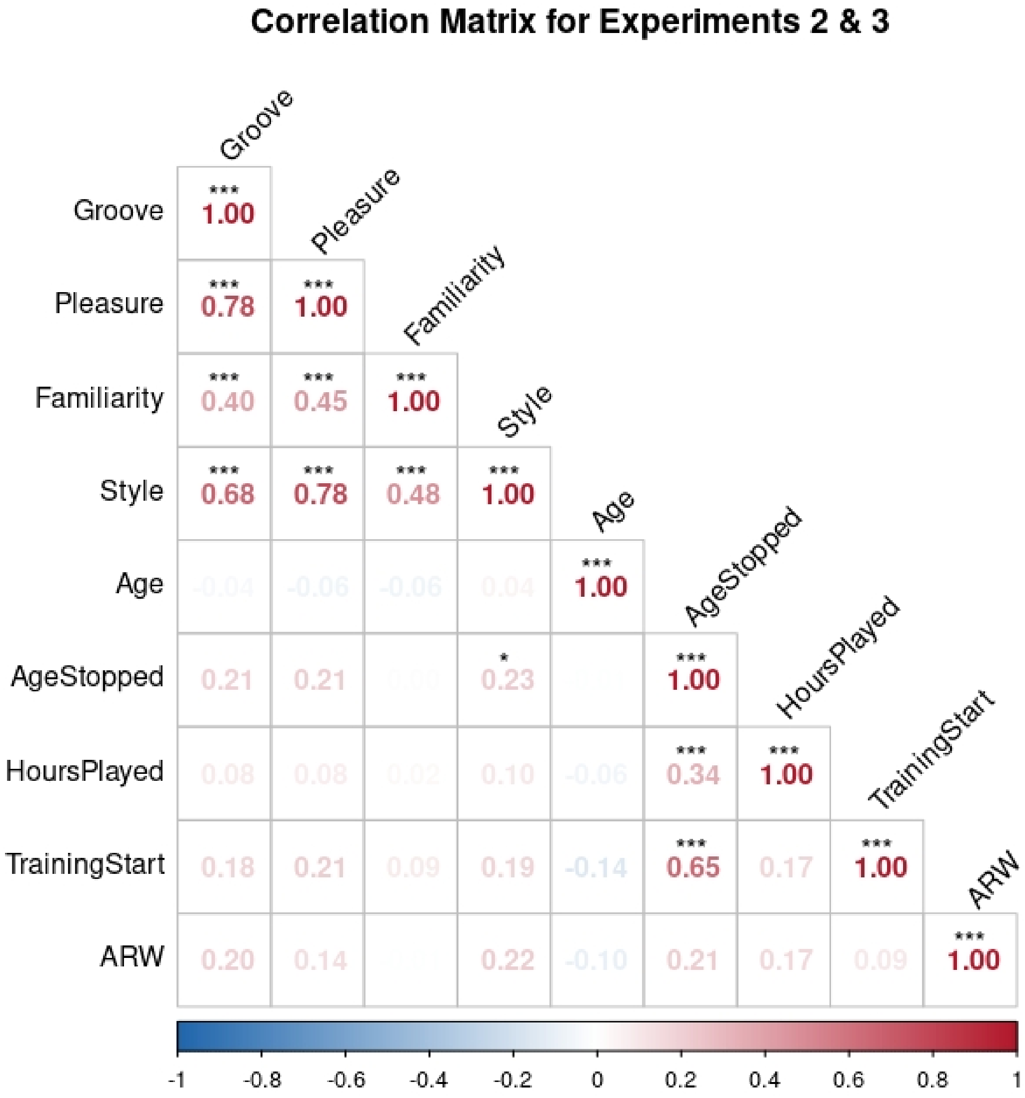

Supplementary Figure 3. Correlation matrix of all measured variables in Experiment 3. “AgeStopped” refers to the age that participants stopped playing music (set to current age if they are still playing music), “HoursPlayed” refers to the reported hours of music playing per week, “TrainingStart” refers to the age that formal musical training started (set to zero if they never started formal musical training), and “ARW” refers to the overall musical reward sensitivity obtained from the Barcelona Musical Reward Questionnaire. Bonferroni-corrected alpha values at $p < 0.05$, $p < 0.01$, and $p < 0.001$ are denoted with single, double, and triple asterisks.

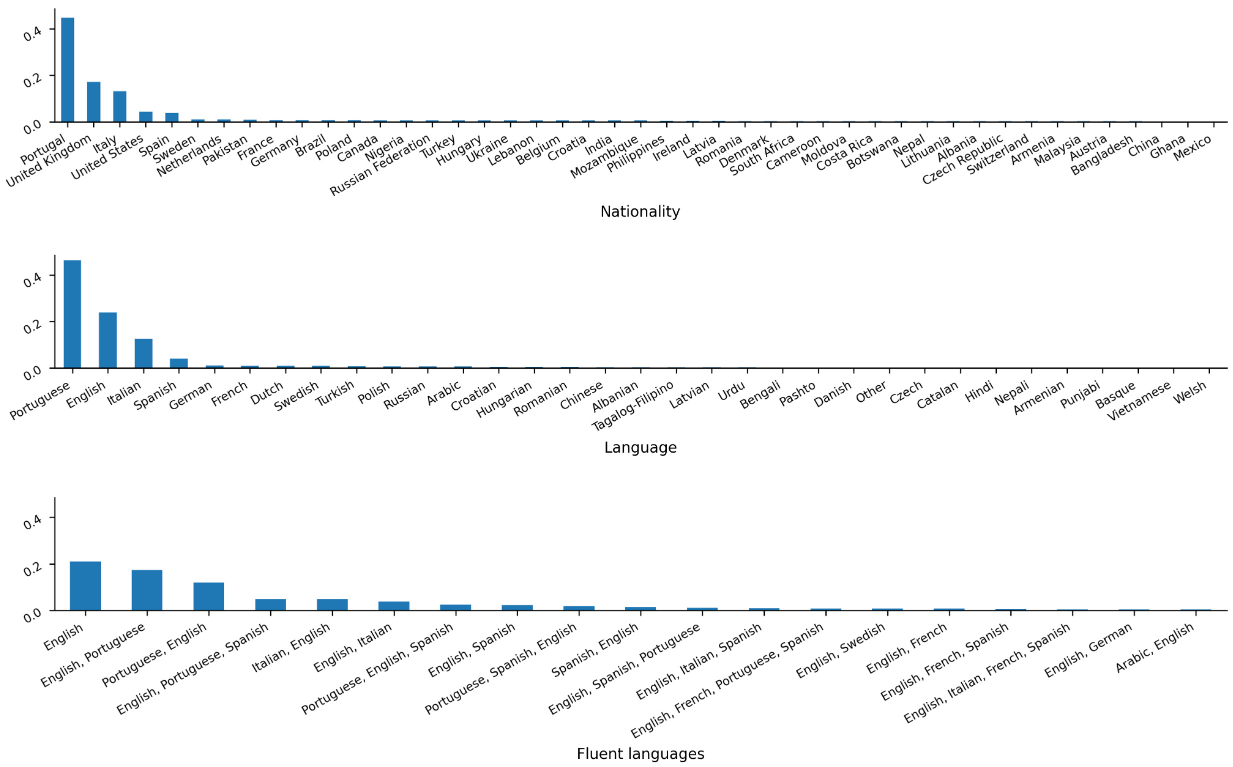

Supplementary Figure 4. Distribution of nationalities, first language, and fluent languages across all three experiments.

Supplementary Analysis 1: Controlling for pulse entropy range

Readers could believe that our results were driven by the 4/4 tracks exhibiting a wider range of pulse entropy values. However, the average pulse entropy value doesn't differ between 4/4 and non-4/4 stimuli in either frequentist or Bayesian independent samples *t*-tests ($t(48.49) = 0.85605$, $p = 0.396$, BF = 0.366). Further, to assess the possible effect of differences in the range of the pulse entropy values, we removed three low entropy excerpts and one high-entropy excerpt from the 4/4 stimulus set to produce an equivalent range to the non-4/4 set. When we re-run the urge to move and pleasure analyses with this truncated 4/4 set, the best models still support an interaction between pulse entropy and meter (Urge to Move: BF = 6.24×10^6; Pleasure: BF = 3.08×10^5). For Urge to Move, this interaction was driven by the quadratic term for pulse entropy where, for the non-4/4 tracks, the same positive quadratic relationship was observed ($\beta_1 = 207.61$, 95% CI [169.58, 247.30], BF = 1.57×10^8) while there was only a negative linear term for the 4/4 tracks ($\beta_1 = -142.31$, 95% CI [-185.37, -100.69], BF = 956.39). For pleasure, the interaction was driven by the linear term of pulse entropy where, for the non-4/4 tracks, the same null effect was observed (BF = 0.608 for the linear term, BF = 0.042 for the quadratic term) and only a negative linear relationship was observed for the 4/4 tracks ($\beta_1 = -145.37$, 95% CI [-180.56, -109.07], BF = 1.96×10^5). In summary, truncating the range of pulse

entropy only seems to trim off the decrease in groove for lower complexity stimuli in 4/4 while maintaining elevated groove for mid-complexity stimuli.

Supplementary Analysis 2: Controlling for tempo

While all musical clips were restricted to be within a narrow range of tempi, it's possible that, on average, the non-4/4 clips may have been faster or slower than the 4/4 clips. However, the average tempo did not statistically differ between the two meter conditions ($t(53.162) = 0.067$, $p = 0.947$, BF = 0.270). Because bpm markings are somewhat subjective, we calculated the event density of each clip with the MIR Toolbox and tested whether it differed between the two meter conditions. Both Welch's two sample and Bayesian independent *t*-tests revealed that average event density did not statistically differ between the two meter conditions ($t(50.963) = 1.1201$, $p = 0.2679$, BF = 0.455). Thus, it seems unlikely that our results were confounded by tempo or event density.

Supplementary Analysis 3: Potential effects of musical training

It's possible that only very highly trained musicians would have greater experience with a broader range of music with a wider variety of metric structures (Jacoby & McDermott, 2017). To explicitly assess this possibility, we split our sample from Experiments 2 and 3 into those with more than 10 years of music playing (N = 49) and those with 0 years of playing (N = 72). The results between the two groups did not differ significantly (see plots below), confirming why musician-related covariates did not improve model fits or interact significantly with our variables of interest. Future work addressing this issue could examine jazz musicians who are more specifically trained in complex meters to further confirm the contribution of top-down effects.

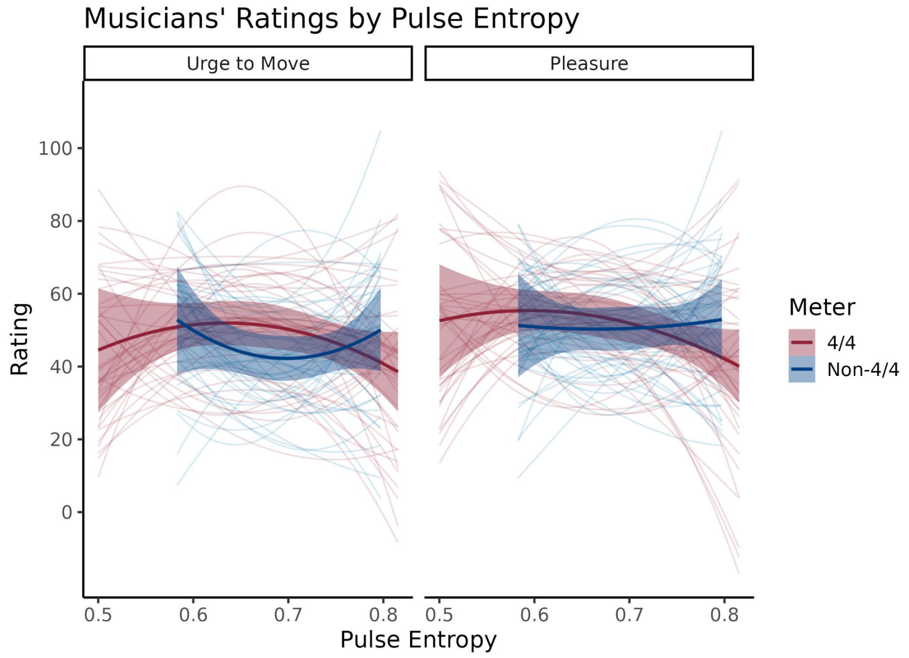

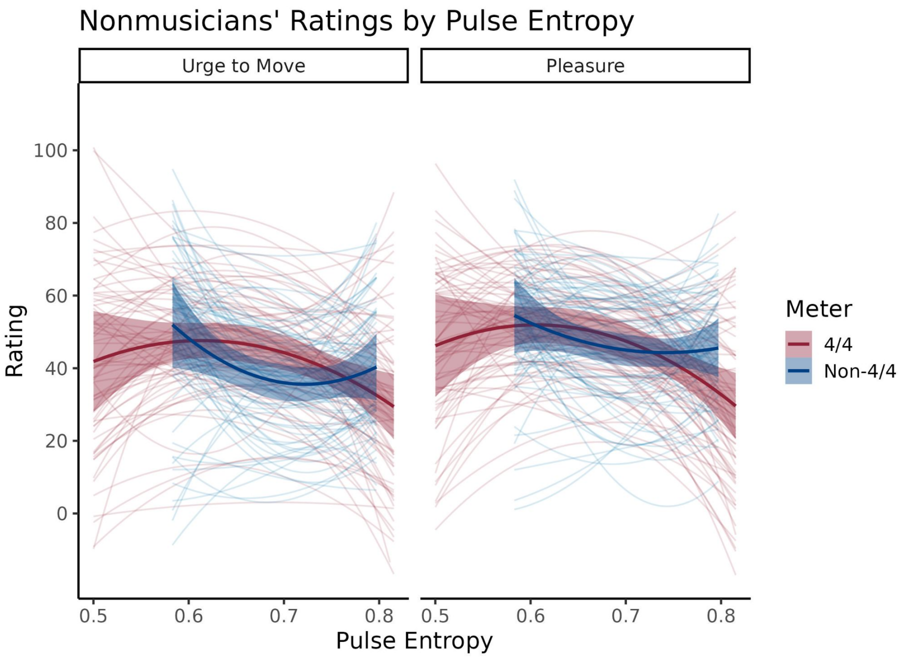

Nonmusicians' Ratings by Pulse Entropy
Urge to Move
Pleasure
Rating
100
80
60
40
20
0
0.5
0.6
0.7
0.8
Pulse Entropy
Meter
4/4
Non-4/4
